# Assessment of Therapeutic Benefit and Option Strategy on Intravesical Instillation for Preventing Bladder Cancer Recurrence after Radical Nephroureterectomy in Patients with Upper Urinary Tract Urothelial Carcinoma

**DOI:** 10.1155/2022/1755368

**Published:** 2022-05-30

**Authors:** Bo Fan, Qiliang Teng, Min Sun, Yingzi Wang, Yutong Wang, Zhe Lin, Yuchao Wang, Xu Duan, Liren Zhang, Tingyu Chen, Sishan Chen, Yu Tai, Ce Zhang, Xishuang Song, Zhiyu Liu

**Affiliations:** ^1^Department of Urology, Second Affiliated Hospital of Dalian Medical University, Dalian 116023, China; ^2^Department of General Surgery, Taihe Hospital, Hubei University of Medicine, Shiyan 442099, China; ^3^Department of International Medical, Second Affiliated Hospital of Dalian Medical University, Dalian 116023, China; ^4^First Clinical College, Dalian Medical University, Dalian 116000, China; ^5^Ethics Committee, Second Affiliated Hospital of Dalian Medical University, Dalian 116023, China; ^6^Second Clinical College, Dalian Medical University, Dalian 116000, China; ^7^Department of Anesthesia, Dalian Medical University, Dalian 116000, China; ^8^Director of the Development Planning and Quality Management Department, Second Affiliated Hospital of Dalian Medical University, Dalian 116023, China; ^9^Department of Urology, First Affiliated Hospital of Dalian Medical University, Dalian 116011, China

## Abstract

**Objective:**

Upper urinary tract urothelial carcinoma (UUT-UC) is a very aggressive disease, characterized by 22%–50% of patients suffering from subsequent bladder recurrence after radical nephroureterectomy (RNU). Although the therapy of intravesical instillation is reported to be effective in preventing bladder recurrence, no study had been reported in Northeast China. The findings relating to the clinical effectiveness of intravesical instillation after RNU are somewhat controversial, and the best efficacy and least adverse effects of instillation drugs have not been widely accepted. Here, we aimed at evaluating the efficacy of intravesical instillation for the prevention intravesical recurrence systematically.

**Methods:**

In this retrospective cohort study, from October 2006 to September 2017, 158 UUT-UC patients underwent RNU were divided into 4 groups: epirubicin (EPB) instillation group, hydroxycamptothecin (HCPT) instillation group, bacillus Calmette–Guerin (BCG) instillation group, and noninstillation group. Cox univariate and multivariate analyses were employed to identify the risk factors for intravesical recurrence-free survival (IVRFS). The nomogram model was also applied to predict patient outcomes. Subsequently, to evaluate the clinical significance of intravesical instillation comprehensively, several databases including PubMed, Ovid, and Embase were searched and data from published studies with our results were combined by direct meta-analysis. Moreover, a network meta-analysis comparing instillation therapies was conducted to evaluate the clinical efficacy of different instillation drugs.

**Results:**

In our retrospective cohort study, the Kaplan–Meier survival curve demonstrated noninstillation groups were associated with worsened IVRFS. Meanwhile, multivariate analysis indicated that intravesical instillation was independent protective factors for IVRFS (hazard ratio [HR] = 0.731). Moreover, calibration plots, receiver operating characteristic (ROC) curves, area under the curve (AUC) values, and the C-index showed the priority of nomogram's predictive accuracy. Next, direct meta-analysis including 19 studies showed that intravesical instillation could prevent the recurrence of bladder cancer with a pooled risk ratio (RR) estimate of 0.53. Subgroup analysis by study type, year of intravesical recurrence, first instillation time, and instillation times also confirmed the robustness of the results. Moreover, intraoperative instillation was associated with a decrease in the risk of bladder recurrence compared with postoperative instillation. Then, a network meta-analysis including 7 studies indicated that pirarubicin (THP) (surface under the cumulative ranking curve [SUCRA] = 89.2%) is the most effective therapy to reduce the risk of bladder recurrence, followed by BCG (SUCRA = 83.5%), mitomycin C (MMC) (SUCRA = 53.6%), EPB (SUCRA = 52.6%), and HCPT (SUCRA = 5.1%) after the analysis of the value ranking.

**Conclusions:**

A maintenance schedule of intravesical instillation prevents the recurrence of bladder cancer after RNU in UUT-UC patients effectively. Large, prospective trials are needed to further confirm its value. Compared with other chemotherapy regimens, THP may be a promising drug with favorable efficacy to prevent bladder recurrence. As included studies had moderate risk of bias, the results of network meta-analysis should be applied with caution.

## 1. Introduction

Upper urinary tract urothelial carcinoma is a rare, latent fatal disease that accounts for nearly 5% of urothelial and 10% of renal tumors [1–4]. Currently, radical nephroureterectomy with bladder cuff removal is the standard treatment [3, 5–8]. Surgery alone can provide sufficient locoregional control only for patients with disease of early stage; however, the overall 5-year survival rate for patients with locally advanced urothelial carcinoma of the renal pelvis and ureter is 0% to 34% after surgery [9–12]. Moreover, urothelial cancer tumors are formed synchronously and/or metachronously in multiple foci throughout the whole urinary tract, which is one of the most essential characteristics of urothelial cancer. Previous studies have indicated that 22–50% of patients undergoing surgical treatment for UUT-UC develop subsequent urinary bladder recurrence [3, 6, 13–20].

Although intravesical instillation is a well-established treatment for preventing intravesical recurrence after the transurethral resection of superficial bladder tumors, there is no consensus on the preventive ability of intravesical instillation to inhibit bladder recurrence after RNU for UUT-UC [15, 18, 21–23]. In 2020, a survey was investigated to explore the use of intravesical instillation in daily practice among European colleagues. Surprisingly, less than half (47%) delivered intravesical instillation after RNU regularly, and 35% ignored the evidence [24]. This may be explained by the limited relevant studies and inconsistent conclusions. O'Brien T et al. demonstrated that the intravesical instillation of MMC could reduce the bladder recurrence rate significantly following nephroureterectomy for UUT-UC [18], which is similar to a randomized controlled trial (RCT) study performed by Ito A et al. in Japan [23]. However, no statistically significant difference was noticed in the rates of bladder recurrence among different intravesical instillation groups and the control group in the studies of Wu et al. [15] and Sakamoto et al. [21] Therefore, in this study, we first determined the clinical significance of intravesical instillation in a retrospective cohort enrolling 158 UUT-UC patients to predict intravesical recurrence-free survival. Then, to comprehensively evaluate the efficacy of intravesical instillation for the prevention of intravesical recurrence after RNU, we performed a meta-analysis with relevant, eligible published research studies on the basis of searching electronic journals. Meanwhile, we conducted subgroup analyses according to the study type, time of the first instillation, instillation times, and yearly recurrence probability to further verify our findings.

Despite this, we also cannot judge which chemotherapy drug and instillation strategies are the most effective. The ODMIT-C trial demonstrated that the intravesical instillation of MMC could reduce the bladder recurrence rate significantly [18]. A prospective randomized phase II study in Japan suggested that the intravesical instillation of THP appears to be effective for preventing bladder recurrence [23]. Another study suggested that no statistically significant difference was found in the bladder recurrence rates among the MMC group, EPB group, and control group [15]. Since network meta-analyses are used to analyze multiple therapies synchronously by integrating direct and indirect evidence, we then conducted network meta-analyses to assess the comparable effectiveness of various instillation regimens.

## 2. Materials and Methods

### 2.1. Retrospective Cohort Study

#### 2.1.1. Patient Assessment

We retrospectively collected the clinical data of patients diagnosed with UUT-UC who underwent RNU with bladder cuff removal at the Second Hospital of Dalian Medical University from October 2006 to September 2017. The inclusion criteria were as follows: (i) patients pathologically diagnosed with primary UUT-UC without the history of bladder cancer; (ii) patients underwent RNU; and (iii) patients had full clinical record and followed-up data. The exclusion criteria are as follows: (i) patients with concomitant bladder cancer or had a previous history of bladder cancer; (ii) patients diagnosed with metastatic systemic disease such as bone metastases, lung metastases, and liver metastases; and (iii) patients receiving neoadjuvant radiotherapy or chemotherapy, systemic chemotherapy, and endoscopic treatments. Clinical information on demographic characteristics, relevant inspection results, and follow-up records was collected. The selection of instillation drugs, including BCG, EPB, and HCPT, mainly depended on the description of the cost of therapy, adverse events during treatment, the voluntary nature of the patient, and surgeons' experience. After excluding patients who stopped intravesical chemotherapy halfway due to adverse events or lost to follow-up, one hundred and fifty-eight patients were retrospectively enrolled in this study cohort. Among them, 41 patients underwent intravesical instillation of 30 mg EPB in 50 ml saline solution after surgery; 33 patients underwent intravesical instillation of 10 mg HCPT in 50 ml saline solution after surgery; 36 patients underwent intravesical instillation of 120 mg BCG in 50 ml saline solution after surgery; and the other patients did not receive preventive instillation after RNU. All instillation groups were initiated two weeks after surgery, and the frequency of instillation was once a week. The solution was retained for at least half an hour. In all, a total of 8 instillations were given over a period of 2 months.

#### 2.1.2. Follow-up Regimen

All patients generally underwent cystoscopy, urinalysis, and cytologic examination per 3 months in the first 2 years, per 6 months in the second year, and annually thereafter to screen for recurrence. Chest/abdominal computed tomography (CT) scans and bone scans were conducted when clinically indicated. Recurrence was identified as pathologic confirmation of urinary tract cancer by cystoscopic biopsy or transurethral resection. The primary endpoint was IVRFS, which was measured from surgery to the date at which recurrence was pathologically confirmed.

#### 2.1.3. Statistical Analysis

The clinicopathological factors affecting bladder recurrence were compared by Student's *t*-test and the *χ*^2^ test for continuous and categorical variables, respectively. The probabilities of IVRFS were determined by Kaplan–Meier survival analysis, and the log-rank test values were applied to assess the differences of statistical significance. The prognostic effects of clinical variables on recurrence were estimated by univariate and multivariate Cox regression analyses. HRs with 95% confidence intervals (CIs) were used to appraise the strength of the individual variables. Statistical analysis was conducted with SPSS 14.0, and *p* < 0.05 represented statistical significance.

Afterwards, a nomogram was constructed to predict 1-, 3-, and 5-year IVRFS by including independent prognostic factors using the rms package in *R* software version 3.51. The concordance index (C-index) was used to estimate the predictive efficiency and distinguishing ability of the nomogram, which mainly gauged the differences between the predicted and actual outcomes. A superior prognostic accuracy for the model is demonstrated by a higher C-index. ROC curves were constructed to determine the sensitivity and specificity of the nomogram, similar to the use of the C-index. The calibration curve was used to indicate the calibration between the observed recurrence and nomogram-predicted recurrence. Furthermore, the nonadherence nomogram was verified by bootstrapping validation (1,000 bootstrap resamples) to adjust a relatively corrected C-index.

### 2.2. Meta-Analysis

#### 2.2.1. Search Strategy

**Figure 1:**
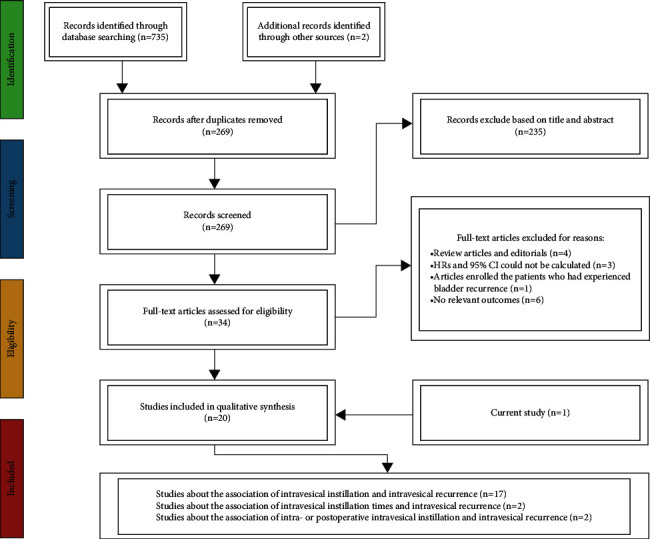
Preferred Reporting Items for Systematic Review and Meta-Analysis (PRISMA) flowchart of the selection of studies.

The PubMed, Google Scholar, Embase, Ovid MEDLINE, and Cochrane Library databases were used to search studies published before December 2021. The following relevant keywords were used: “upper urinary tract,” “urothelial carcinoma,” “bladder recurrence,” “intravesical recurrence,” “intravesical irrigation,” “intravesical instillation,” “intravesical chemotherapy,” “renal pelvis,” and “ureter.” Bibliographies of the retrieved articles were also hand-searched to identify other potentially eligible trials. No filters were applied for the date of publication or language. Our study was performed based on the PRISMA (Preferred Reporting Items for Systematic Review and Meta-Analyses) statement. The PRISMA checklist is shown in Supplementary Table 1. The study has been registered at the PROSPERO register (https://www.crd.york.ac.uk/PROSPERO, registration number: CRD42021285316). A flow diagram of the literature search is shown in Figure 1.

#### 2.2.2. Inclusion and Exclusion Criteria

Studies were eligible when they satisfied the following criteria: (i) patients with UUT-UC were confirmed after RNU; (ii) the intervention group received intravesical instillation with any kind of chemotherapy or BCG; (iii) the association between intravesical chemotherapy and bladder recurrence was reported; and (iv) the RR or HR for intravesical recurrence-free survival rate after RNU was reported or could be calculated. The following exclusion criteria were used: (i) single-arm studies, case reports, letters to the editor without original data, reviews, or commentaries; (ii) duplicated publications for the same author or institute; (iii) failure to provide information on bladder recurrence after RNU; (iv) based on the given information, absence of information on the HR or RR and its standard error; (v) articles evaluating the recurrence-free survival rates after preoperative; and (vi) articles enrolled the patients who had experienced bladder recurrence.

#### 2.2.3. Data Extraction

A meticulous procedure was conducted independently by three investigators (Bo Fan, Qiliang Teng, and Yingzi Wang), who selected potentially related studies according to the predetermined criteria. Any discrepancies in extracting data were assessed by three reviewers (Zhiyu Liu, Xishuang Song, and Guoyu Wu), who checked the resulting extractions. Data collected from the studies included study type, region, year of publication, number of patients, recruitment period, author, age, sex, tumor characteristics, information of intravesical instillation (regimens, first instillation time, instillation times, treatment duration), and the HR/RR and its 95% CI for intravesical recurrence-free survival. IVRFS was regarded as the interval between RNU and the first intravesical recurrence.

#### 2.2.4. Sensitivity Analysis

The leave-one-out approach was adopted to delete individual trials sequentially. The fixed-effects model was conducted after deleting the studies with heterogeneity if the *p* value was greater than 0.05 by the Q-test. The random-effects model was conducted when there was heterogeneity observed after removing the individual study.

#### 2.2.5. Statistical Analysis

*(1) Direct Meta-analysis*. For dichotomous outcomes, the RR with its 95% CI was estimated. The *χ*^2^ test and the Tau^2^ and I^2^ statistics were calculated to assess heterogeneity, estimate the overall/residual heterogeneity, and evaluate the inconsistency percentage, respectively. There was no heterogeneity in studies when the *p* value was more than 0.1 in the *χ*^2^ test. The pooled RRs and corresponding 95% CIs of each study were estimated by fixed-effects models. Otherwise, random-effects models will be conducted. The *I*^2^ value reflected the proportion of the overall difference that was due to heterogeneity instead of sampling errors. An *I*^2^ statistic >50% indicated the presence of high heterogeneity; then, the random-effects model will be carried out. Otherwise, the fixed-effects model was used. The *Z* test was used to assess the significance of the overall effect, and the funnel plot indicated the potential publication bias. The results were defined as significant statistically with two-sided *p* values < 0.05. The direct meta-analysis was conducted by Stata 12.0 (Stata Corporation, College Station, TX, USA).

*(2) Network Meta-analysis (NMA)*. The NMA within a Bayesian framework was performed by synthesizing direct and indirect evidence of intravesical instillation regimens. A net plot was constructed to demonstrate the connection between each intervention and the endpoint [25]. We used the inconsistency factor (IF) to assess the possible sources of inconsistency among trials within direct and indirect effectiveness for the same comparison. IF values with 95% CIs were truncated at zero, indicating no statistically significant deviation [26]. The summary treatment effects on each comparison were estimated as RRs with 95% CIs in a forest plot. In addition, the SUCRA was used to rank the effects of different treatment options [27]. The contribution of each direct comparison to the combined results is shown by a contribution plot [28]. Moreover, we evaluated the deviation in loops of the intravesical instillation network by adopting loop-specific heterogeneity estimates through the method of moments. A comparison-adjusted funnel plot was carried out to assess publication bias. A conclusion of no publication bias was considered when the funnel plots were scattered symmetrically around the zero line [29].

## 3. Results

### 3.1. Baseline Patient Characteristics

We analyzed 158 UUT-UC patients including 88 males and 70 females with a median age at diagnosis of 69 years (range from 59 to 80) from October 2006 to September 2017. The clinicopathological characteristics of patients are presented in Table 1, and no statistically significant differences were observed between the groups in terms of age, tumor laterality, tumor location, tumor focality, vessel tumor embolus, tumor stage, pathologic grade, lymph node status, perineural invasion, or type of surgery except for sex. The median follow-up time was 29.2 months (range from 2.3 to 122.1). Among 158 patients, 63 subsequently had bladder tumor recurrence during follow-up after nephroureterectomy. The intravesical recurrence rate of the noninstillation group was higher than that of the EPB group, HCPT group, and BCG group (60.4% vs. 24.4%, 57.6%, and 13.9%, respectively).

### 3.2. The Effectiveness of Intravesical Instillation for Intravesical Recurrence in UUT-UC Patients

**Figure 2:**
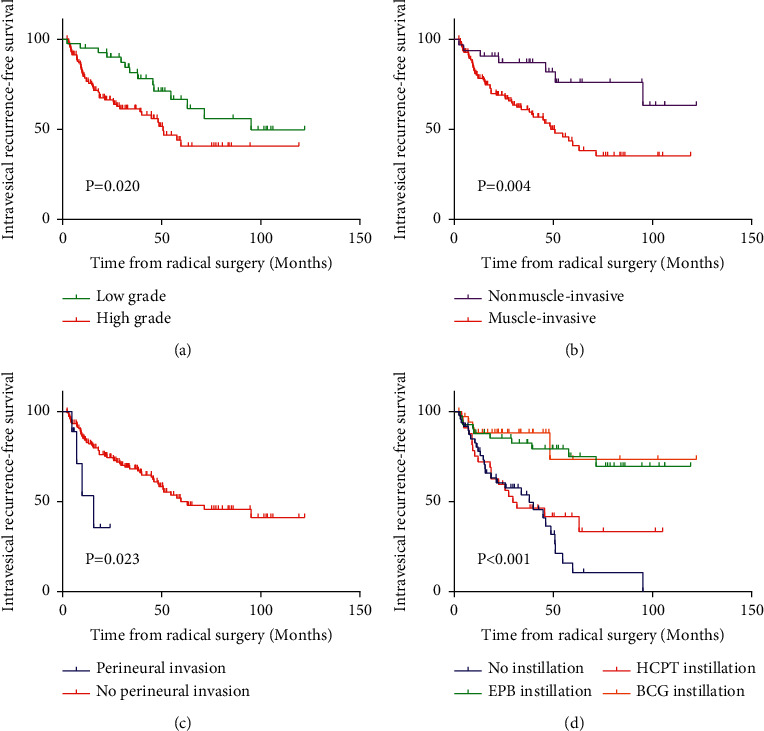
Intravesical recurrence-free survival (IVRFS) curves for the eligible patients by Kaplan–Meier survival analysis and log-rank tests. Intravesical recurrence-free survival for high-grade (red line) and low-grade (green line) patients, demonstrating that patients with low-grade upper urinary tract urothelial carcinoma (UUT-UC) had a significantly better IVRFS than individuals with high-grade disease (a). Intravesical recurrence-free survival for nonmuscle-invasive (purple line) and muscle-invasive (red line) patients, suggesting that muscle invasion was significantly associated with worse IVRFS (b). Intravesical recurrence-free survival for perineural invasion (blue line) and no perineural invasion (red line) patients, showing that perineural invasion was related to worse IVRFS significantly (c). Intravesical recurrence-free survival curves for no instillation (blue line), EPB instillation (green line), HCPT instillation (red line), and BCG instillation (yellow line), illustrating that instillation groups had a significantly better IVRFS than the no-instillation group (d).

Kaplan–Meier survival curves of IVRFS were plotted according to pathological grade, tumor stage, perineural invasion, and intravesical instillation. Lower pathological grade (*p*=0.020), nonmuscular invasive type (*p*=0.004), no perineural invasion (*p*=0.023), and instillation group (*p* < 0.001) achieved a better effect on IVRFS (Figures 2(a)–2(d)). Univariate analysis revealed that intravesical instillation (*p*=0.007), tumor stage (*p*=0.006), perineural invasion (*p*=0.032), and pathological grade (*p*=0.023) were significant prognostic factors for IVRFS. Tumor stage (*p*=0.016), perineural invasion (*p*=0.043), and intravesical instillation (*p*=0.015) were defined as independent prognostic indicators for IVRFS in UUT-UC patients by multivariate analysis (Table 2).

**Figure 3:**
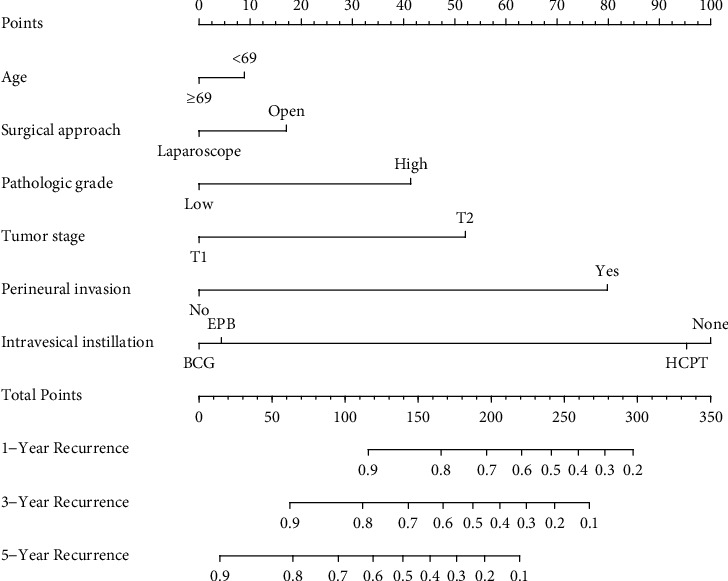
1-, 3-, and 5-year intravesical recurrence-free survival (IVRFS)-associated nomogram model. The nomogram of IVRFS was based on six factors and established through Cox regression analysis. Abbreviations: EPB, epirubicin; HCPT, hydroxycamptothecin; BCG, bacillus Calmette–Guerin.

**Figure 4:**
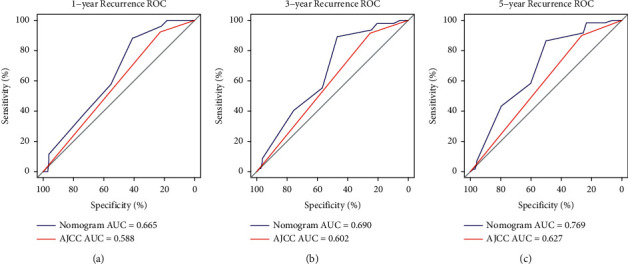
The receiver operating characteristic (ROC) curves of 1-, 3-, and 5-year intravesical recurrence-free survival (IVRFS) after nephroureterectomy. The ROC curve of 1-year IVRFS (a). The ROC curve of 3-year IVRFS (b). The ROC curve of 5-year IVRFS (c). The blue lines represent nomogram-predicted IVRFS, and the red lines represent AJCC-predicted IVRFS.

The nomogram model for predicting the 1-, 3-, and 5-year IVRFS rates of individual UUT-UC patients was constructed with three independent prognostic indicators in conjunction with age, surgical approach, and pathological grade (Figure 3). Compared with AJCC models, the ROC curves of 1-, 3-, and 5-year IVRFS nomograms revealed better discrimination efficacy. The AUCs of the 1-, 3-, and 5-year IVRFS AJCC models and the nomogram models were equal to 0.588 vs. 0.665, 0.602 vs. 0.690, and 0.627 vs. 0.769, respectively (Figure 4). Good discrimination was also obtained for the bootstrapping method, and the C-index of the nomogram model was equal to 0.704.

**Figure 5:**
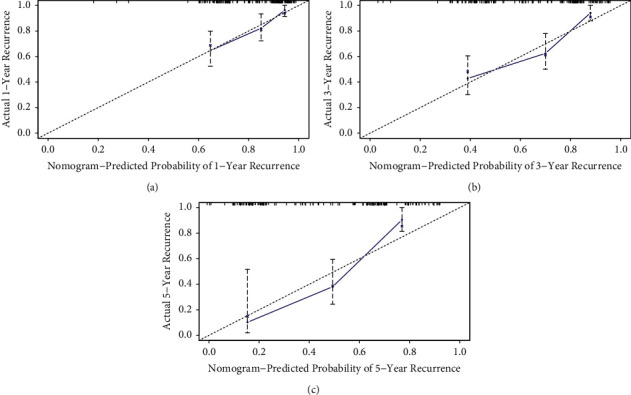
Calibration curves for predicting intravesical recurrence-free survival (IVRFS) after nephroureterectomy. The 1-year IVRFS calibration curve (a). The 3-year IVRFS calibration curve (b). The 5-year IVRFS calibration curve (c). The black dotted line refers to the optimal nomogram; circles represent the accuracy of prediction; X demonstrates the bootstrap-revised estimates; and erected lines indicate the 95% confidence intervals (CIs).

The included factors were selected according to multivariate analysis and clinical experience. The nomogram was internally validated. The C-index for IVRFS prediction of the nomograms was 0.737 (95% CI, 0.672–0.801). Furthermore, the relatively corrected C-index of 0.704 was calculated by bootstrapping validation (1,000 bootstrap resamples). Finally, the calibration plots of the nomogram presented a relatively optimal consistency between the actual observations and the predictions (Figure 5).

### 3.3. Meta-Analysis of the Correlation between Intravesical Instillation and Intravesical Recurrence in UUT-UC Patients

Supplementary Table 2 and Supplementary Table 3 show individual data on characteristics of the included studies and patient population.

#### 3.3.1. Primary Outcome

**Figure 6:**
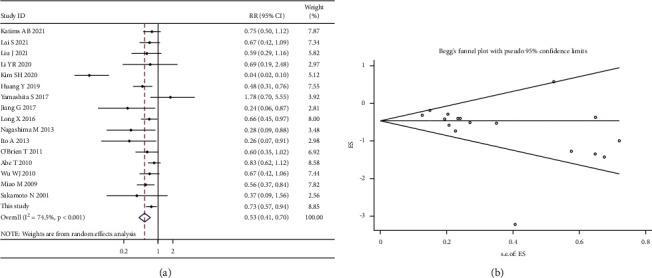
Overall meta-analysis for intravesical recurrence. Forest and funnel plots for the effect of intravesical instillation on bladder recurrence (a, b). Every square was displayed as a trial, and the range of every square is proportional to the accuracy of the average treatment effectiveness of the study. The 95% confidence interval (CI) for the treatment effectiveness of each study was indicated by the horizontal line. The center of the block is the mean treatment effectiveness across trials, and the width of the block represents its 95% CI.

**Figure 7:**
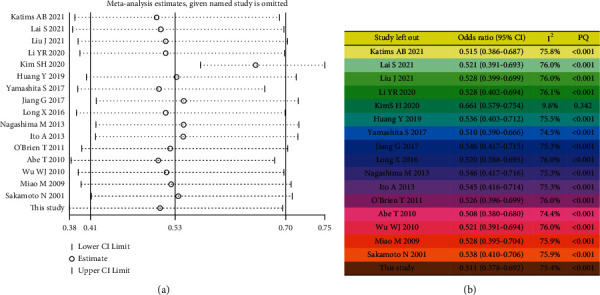
The leave-one-out sensitivity analysis for overall analysis. The forest plot table demonstrates the effects of heterogeneity of individual study (a, b).

All 19 trials reported intravesical instillation in UUT-UC patients. Since there was significant heterogeneity overall (*χ*^2^ = 62.66; *p* value for heterogeneity < 0.001; *I*^2^ = 74.5%), the random-effects model was adopted to measure each study. The pooled results of these trials showed that the use of intravesical instillation lowered the intravesical recurrence rate (RR, 0.53; 95% CI, 0.41–0.70; *p* < 0.001; Figure 6(a)). As determined by Begg's test and Egger's test, the result exhibited a low probability of publication bias (Egger's test, *p*=0.066; Begg's test, *z* value = 2.43) (Figure 6(b)). Then, the sequential sensitivity analysis was performed to assess heterogeneity in individual study. By removing any of studies, there was no obvious individual heterogeneity, indicating that the random-effects model with pooled data had moderate reliability. In the leave-one-study-out sensitivity analysis, the complete *I*^2^ statistical values and *p* values by the Q-test are summarized in Figure 7.

#### 3.3.2. Secondary Outcomes

**Figure 8:**
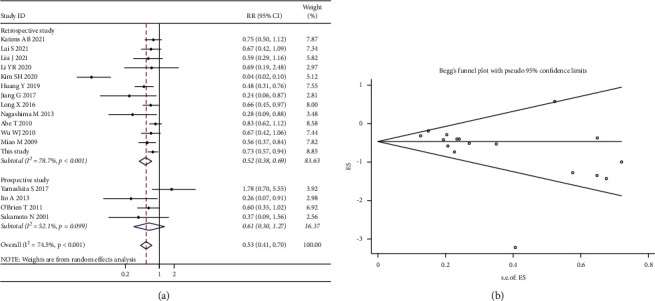
Forest and funnel plots of the effect of intravesical instillation on bladder recurrence by subgroup analysis according to the study type (a, b).

*(1) Subgroup Analysis by Study Type*. Subgroup analysis was performed by the random-effects model, since significant heterogeneity was found across studies (*χ*^2^ = 62.66; *p* value for heterogeneity < 0.001; I^2^ = 74.50%). The aggregated results demonstrated the incidence of intravesical recurrence in the instillation group was lower than that in the control group in both the retrospective study subgroup (RR, 0.52; 95% CI, 0.38–0.69; *p* < 0.001) and the prospective study subgroup (RR, 0.61; 95% CI, 0.30–1.27; *p*=0.188) (Figure 8(a)). Publication bias was indicated by the funnel plot (Figure 8(b)) and formal statistical analysis (Egger's test, *p* < 0.001; Begg's test, *z* value = 3.07).

**Figure 9:**
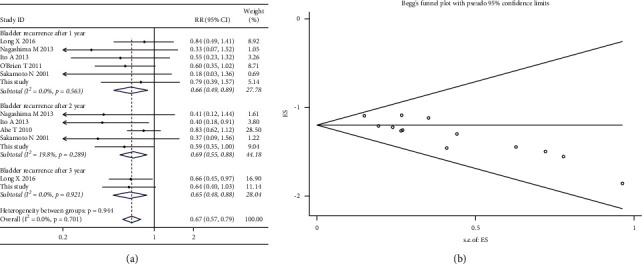
Forest and funnel plots of the effect of intravesical instillation on bladder recurrence by subgroup analysis according to the timing of recurrence (a, b).

*(2) Subgroup Analysis by Year of Intravesical Recurrence*. When the seven included studies were pooled in subgroup analyses, the fixed-effects model was carried out, since no heterogeneity was seen (*χ*^2^ = 9.02; *p* value for heterogeneity = 0.701; I^2^ = 0.0%). Compared with the noninstillation group, the instillation group was associated with decreases in intravesical recurrence after 1 year (RR, 0.66; 95% CI, 0.49–0.89; *p*=0.007), 2 years (RR, 0.69; 95% CI, 0.55–0.88; *p*=0.002), and 3 years (RR, 0.65; 95% CI, 0.48–0.88; *p*=0.005) (shown in Figure 9(a)). Publication bias was indicated by the funnel plot (Figure 9(b)) and formal statistical analysis (Egger's test, *p*=0.001; Begg's test, *z* value = 3.11).

**Figure 10:**
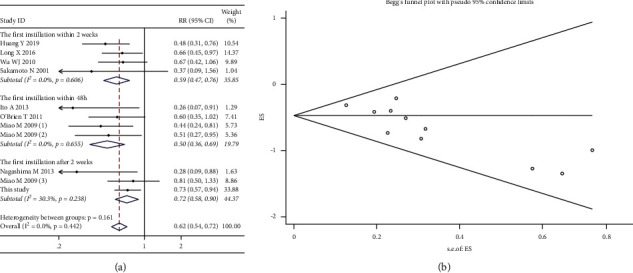
Forest and funnel plots of the effect of intravesical instillation on bladder recurrence by subgroup analysis according to the first instillation time (a, b).

*(3) Subgroup Analysis by The First Instillation Time Postoperatively*. Nine included studies were pooled in subgroup analyses, and the fixed-effects model was conducted, since no heterogeneity was seen (*χ*^2^ = 9.98; *p* value for heterogeneity = 0.442; I^2^ = 0.0%). The results demonstrated that the risk of intravesical recurrence in the instillation group was significantly lower than that in the control group in the first instillation within 48-h subgroup (RR, 0.50; 95% CI, 0.36–0.69; *p* < 0.001), the first instillation within 2-week subgroup (RR, 0.59; 95% CI, 0.47–0.76; *P* < 0.001), and the first instillation after 2-week subgroup (RR, 0.72; 95% CI, 0.58–0.90; *p*=0.003) (Figure 10(a)). Publication bias was indicated by the funnel plot (Figure 10(b)) and formal statistical tests (Egger's test, *p*=0.004; Begg's test, *z* value = 2.02).

**Figure 11:**
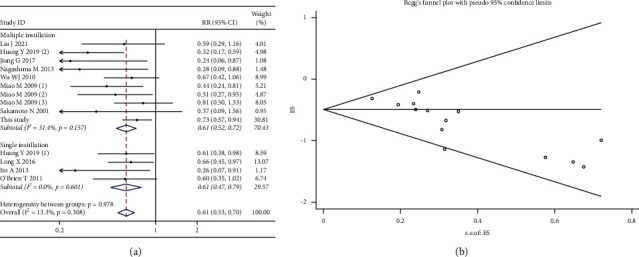
Forest and funnel plots of the effect of intravesical instillation on bladder recurrence by subgroup analysis according to the instillation times (a, b).

*(4) Subgroup Analysis by the Instillation Times.* Twelve included studies were pooled in subgroup analyses, and the fixed-effects model was used, since no heterogeneity was seen (*χ*^2^ = 14.99; *p* value for heterogeneity = 0.308; I^2^ = 13.3%). The results suggested that both the single (RR, 0.61; 95% CI, 0.47–0.79; *p* < 0.001) and multiple (RR, 0.61; 95% CI, 0.52–0.72; *p* < 0.001) instillations group were associated with the reduction in the intravesical recurrence rate (Figure 11(a)). Publication bias was indicated by the funnel plot (Figure 11(b)) and formal statistical tests (Egger's test, *p* < 0.001; Begg's test, *z* value = 3.07).

### 3.4. Meta-Analysis of the Correlation between Intravesical Instillation times and Intravesical Recurrence in UUT-UC Patients

**Figure 12:**
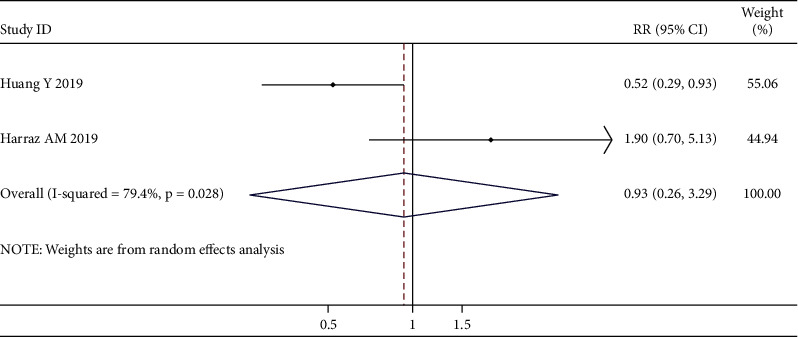
Overall meta-analysis for intravesical recurrence. Forest and funnel plots for the effect of single or multiple instillations on bladder recurrence (a, b).

Figure 12 shows two trials reporting data on the efficiency of single or multiple instillations for the prevention of bladder recurrence. Since significant heterogeneity was discovered, the random-effects model was used (*χ*^2^ = 4.84; *p* value for heterogeneity = 0.028; I^2^ = 79.4%). For the secondary outcomes, no significant differences were observed between the single-instillation group and the multiple-instillation group (RR, 0.93; 95% CI, 0.26–3.29; *p*=0.912).

### 3.5. Meta-Analysis of the Correlation between Intra- or Postoperative Intravesical Instillation and Intravesical Recurrence in UUT-UC Patients

**Figure 13:**
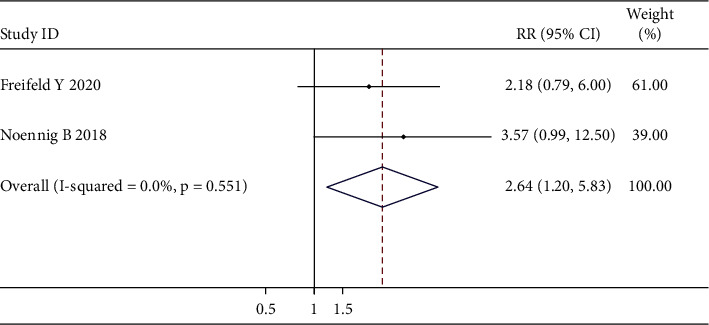
Overall meta-analysis for intravesical recurrence. Forest and funnel plots of the effect of intra- or postoperative instillation on bladder recurrence (a, b).

Figure 13 shows two trials reporting data on the efficiency of intra- or postoperative instillation for the preventing of bladder cancer recurrence. The fixed-effects model was conducted, since no heterogeneity was seen (*χ*^2^ = 0.35; *p* value for heterogeneity = 0.551; I^2^ = 0.0%). For the secondary outcomes, the incidence of intravesical recurrence in the intraoperative instillation group was significantly lower than that in the postoperative instillation group (RR, 2.64; 95% CI, 1.20–5.83; *p*=0.016).

### 3.6. Bayesian Framework Network Meta-Analysis

#### 3.6.1. Network and Contribution

**Figure 14:**
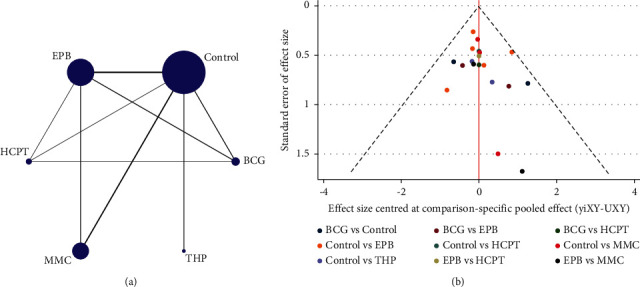
Network plot of different treatment comparisons and comparison-adjusted funnel plot of the network meta-analysis (NMA) (a, b). The thickness of lines is correlated with the number of studies, and the extent of dots demonstrates the sample size of patients. The red line indicates the null hypothesis, and there is no difference between the study-specific effect size and the respective comparison-specific pooled effect estimates. Various colors refer to different comparisons. Abbreviations: EPB, epirubicin; HCPT, hydroxycamptothecin; MMC, mitomycin C; THP, pirarubicin; BCG, bacillus Calmette–Guerin; control, no-instillation group.

**Figure 15:**
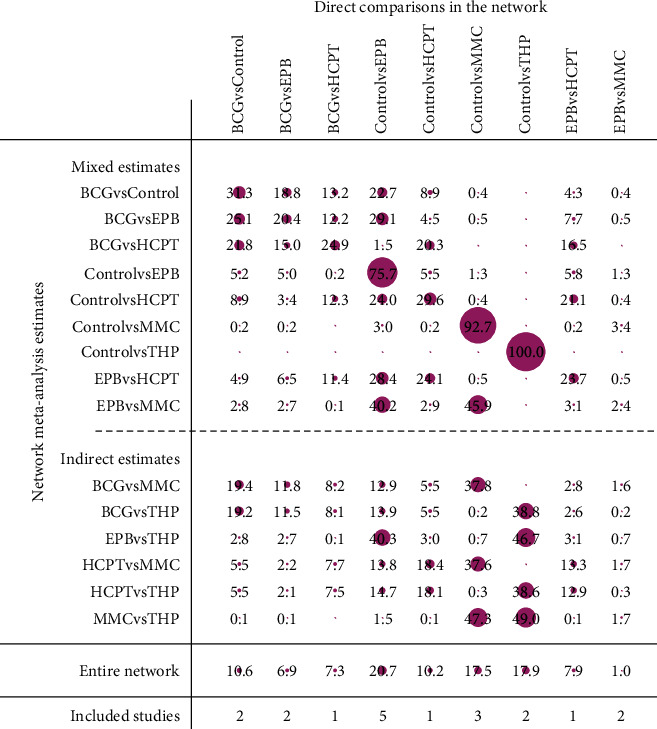
Contribution plot of the enrolled studies. The rows represent all probable pairwise comparisons, and the columns represent the direct comparisons.

To analyze the therapeutic effect of different drugs for bladder instillation, an NMA was performed after including nine studies on the EPB instillation group, HCPT instillation group, THP instillation group, MMC instillation group, and BCG instillation group. Among them, EPB instillation was the most studied treatment. In Figure 14(a), the thickness of the lines correlates with the number of studies, and the scope of the dots represents the trial size in terms of patients. Moreover, although there was just one comparison of THP instillation and its trial size was relatively small, the number of trials with the THP instillation group was sufficient. The HCPT instillation group consisted of three comparisons, and its trial size was smaller than that of the EPB instillation group and BCG instillation group. A contribution plot was generated for each direct comparison (Figure 15). Among these, the control group vs. THP instillation group was informed by direct comparison alone, eight comparisons were informed by mixed evidence, and six comparisons were informed by indirect evidence alone. According to the overall contribution of the network, the control group vs. EPB instillation group (20.7%) had the most influential tendencies. The EPB instillation group vs. MMC instillation group (1.0%) had the smallest impact on the whole analysis.

#### 3.6.2. Network Comparisons and Ranks

**Figure 16:**
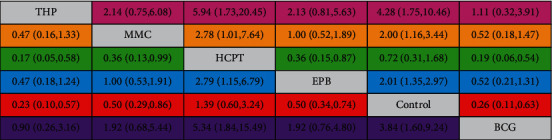
Confidence interval plot of the network analysis. The confidence interval of overall odds ratios with every comparison is shown by each square. The column treatment is compared with the row treatment.

**Figure 17:**
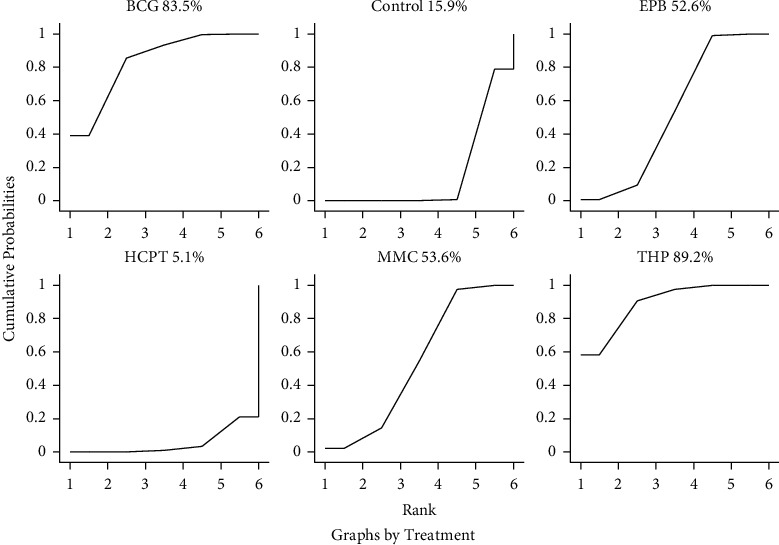
Surface under the cumulative ranking curves of treatments among different intravesical chemotherapy regimens.

The estimated treatment effect and its 95% CI for all comparisons were determined (Figure 16). Of note, for every comparison with the confidence interval, more studies are needed for more apparently significant results. The results demonstrated an impressive tendency in that the THP and BCG instillation groups were more favorable in preventing the incidence of intravesical recurrence than the other instillation groups, while HCPT instillation seemed to be less effective than the instillation of other drugs. The six instillation groups' estimated relative rankings of cumulative probabilities of IVRFS are plotted in Figure 16. The SUCRA value rankings of IVRFS were as follows: THP instillation group (89.2%), BCG instillation group (83.5%), MMC instillation group (53.6%), EPB instillation group (52.6%), and HCPT instillation group (5.1%) (Figure 17). Therefore, THP was a more recommended intravesical treatment in UUT-UC patients after surgery.

#### 3.6.3. Inconsistency Analysis and Publication Bias

**Figure 18:**
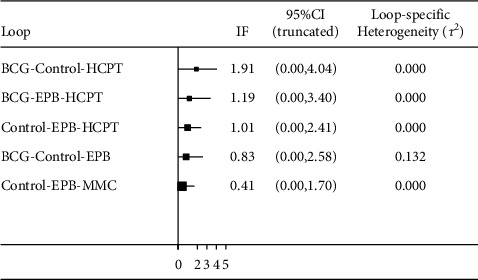
Inconsistency test. Inconsistency indicates the variations between indirect and direct effects estimated for the same comparison. Abbreviation: IF, inconsistency factor.

Loop-specific sensitivity analysis was performed to assess the consistency of the NMA. This NMA was composed of five triangular loops, including BCG-Control-HCPT (B-C-H), BCG-EPB-HCPT (B-E-H), Control-EPB-HCPT (C-E-H), BCG-Control-EPB (B-C-E), and Control-EPB-MMC (C-E-M). All loops revealed no evidence of significant inconsistency (B-C-H, IF = 1.91, 95% CI, 0.00–4.04; B-E-H, IF = 1.19, 95% CI, 0.00–3.40; C-E-H, IF = 1.01, 95% CI, 0.00–2.41; B-C-E, IF = 0.83, 95% CI, 0.00–2.58; C-E-M, IF = 0.41, 95% CI, 0.00–1.70) (Figure 18). The funnel plot for NMA is shown in Figure 14(b). The enrolled studies were distributed symmetrically, demonstrating that this NMA had no significant publication bias(b)

## 4. Discussion

Although radical nephroureterectomy with the removal of an ipsilateral bladder cuff is the most widely recognized treatment for UUT-UC, 25% to 69% of postoperative patients suffer from subsequent intravesical recurrence of bladder carcinoma, which is one of the predominant concerns regarding prognosis [30–34].

Some studies have found that positive urine cytology was an independent predictor for tumor recurrence [35], and the proximity of the site of bladder mucosal injury and intravesical recurrence locations confirmed the “tumor seeding” theory [36]. Multifocal tumors appear from dispersed viable cancer cells and are therefore monoclonal, transforming by either intraluminal seeding or intraepithelial migration, particularly during surgery [37]. In our study, of the 158 patients, 63 subsequently had bladder tumors after nephroureterectomy during follow-up, with an intravesical recurrence rate of 39.8%, which favors the theory of monoclonal origin by intraluminal seeding. Interestingly, Huang et al. proposed that dispersed viable intraluminal cancer cells are not totally eliminated by a single instillation of chemotherapy, and multiple instillations were more efficient, which supported the theory of “field change” [38]. The whole urothelium is exposed to a variety of carcinogenic insults that can lead to malignancy and multifocal tumors, subsequently derived from separate clones of transformed cells [37].

Reviewing past studies, some prognostic factors have been investigated to identify UUT-UC patients after surgery if they are at risk of bladder recurrence, and urologists then stratify these risks. Subsequently, urologists will schedule an individual and stringent follow-up regimen according to the identified prognostic factors, including ureteral location, multifocality, and invasive pT stage. Moreover, Pieras et al. reported that UUT-UC patients with a tumor size >4 cm had a higher rate of intravesical recurrence [39]. Recent studies revealed that ureterorenoscopy for UUT-UC patients who underwent RNU did not prevent bladder recurrence, as UUT-UC implantation of the bladder in cancer cells may arise after ureterorenoscopy, supporting the intraluminal seeding hypothesis [40–42]. At the genetic or molecular biology level, Huang Y et al. confirmed that high expression levels of AIB1 (amplified in breast cancer 1) and EIF5A2 (eukaryotic initiation Factor 5A2) were individual predictive factors for bladder recurrence after RNU, and they thought that this information could help urologists to stratify patients to determine those who would benefit from postoperative intravesical chemotherapy [43]. In our study, multivariate analysis suggested that tumor stage (*p*=0.016), perineural invasion (*p*=0.043), and intravesical instillation (*p*=0.015) were independent prognostic indicators of IVRFS in UUT-UC patients (Table 2).

In this study, we found that intravesical instillation could significantly reduce the bladder recurrence rate of patients who underwent RNU. However, given the few relevant investigations, no consensus has been reached. In a single-institutional study of 320 patients, Long et al. proved that the incidence rates of bladder recurrence were significantly decreased by intravesical chemotherapy [44]. Similarly, in a prospective, randomized trial of 220 patients, O'Brien T et al. suggested that the incidence of bladder recurrence after RNU can be lowered by intravesical chemotherapy [18]. In contrast, Sakamoto et al. did not confirm intravesical chemotherapy as a prognostic factor of bladder recurrence, although a trend was observed [21]. In a long-term retrospective study of 196 patients, Wu et al. also found that the incidence of bladder recurrence was higher in the control group than in the MMC and EPB groups, but there was no significant difference [15]. Hence, on the basis of searching PubMed, Google Scholar, Ovid MEDLINE, Embase, and the Cochrane Library, we combined 19 related studies that compared the difference in bladder recurrence incidence between an instillation group and a noninstillation group and performed a meta-analysis. Our results suggested that the RR was 0.53, and the 95% CI ranged from 0.41 to 0.70, demonstrating that intravesical instillation decreased the risk of bladder recurrence by 47%, with a 95% CI ranging from a decrease of 30% to a decrease of 59%. The earliest meta-analysis about intravesical chemotherapy for the preventing of bladder cancer recurrence after surgery for UUT-UC was published by Fang et al. in 2013 [45]. Two years later, one meta-analysis [46] performing subgroup analysis stratified by study type and another meta-analysis [47] performing subgroup analysis by different starting times and cycles of instillations were published separately, which helped to build a comprehensive and systematic understanding of prophylactic bladder instillation. However, as a study of O'Brien T et al. was included in the above meta-analyses, during data extraction, patients analyzed by intention to treat from patients analyzed per protocol were confused by patients in the instillation group who did not receive it, patients in the control group who receive intravesical chemotherapy and patients who had incomplete submitted data during follow-up [18]. Moreover, a study in which some patients in the intervention group received preoperative intravesical instillation of BCG was included in the meta-analysis by Yuan et al [46]. This may interfere with or influence the quality of their conclusion because of the uncertainty of confounding factors, which may make the results less objective for guiding clinical decisions about drug options.

Although our meta-analysis demonstrated that intravesical instillation can effectively prevent the recurrence of bladder cancer after RNU, the details of the implementation of the instillation regimens are worthy of consideration and discussion. (1). When should clinicians recommend the first intravesical instillation for UUT-UC patients after RNU? In our institution, the recurrence rate of instillation group was 30.9% under the first instillation one month after surgery to prevent the aggravation of bladder spasm, which interfered with the healing of the wound after removal of bladder cuff. As first instillation was started within 48 hours in another study by Ito et al. [23], the incidence of bladder recurrence of the instillation group was reported as 16.9%. After evidence-based analysis, subgroup analysis according to the first instillation time showed that the first instillation within 48 h (RR, 0.50; 95% CI, 0.36–0.69; *p* <  0.001) subgroup may had better preventive effect on bladder cancer recurrence than the first instillation within or after 2 weeks subgroups (RR, 0.59; 95% CI, 0.47–0.76; *p* <  0.001; RR, 0.72; 95% CI, 0.58–0.90; *p*=0.003), which was consistent with those of the study of Wu et al. [47]. Studies have proposed that intraluminal seeding is an important factor leading to the implantation of cancer cells within 24 hours, which is also the basis for the application of a single dose of intravesical instillation after surgery [48]. Therefore, an earlier first instillation time may be recommended for better prophylactic efficacy in preventing intravesical recurrence. (2). How many courses of intravesical instillation should clinicians recommend to UUT-UC patients? In our study, patients of the instillation group underwent intravesical instillation 8 times weekly after surgery. Hwang EC et al. found that single-dose intravesical chemotherapy instillation postoperatively may reduce the incidence of bladder cancer recurrence (HR, 0.51; 95% CI, 0.32–0.82) [49]. However, a multicenter study performed by O'Brien T et al. conducted a single postoperative intravesical dose of MMC [18]. After including 12 related studies, the subgroup analysis stratified by instillation times were conducted and the result revealed that, compared with the respective control group, both intravesical single instillation (RR, 0.61; 95% CI, 0.47–0.79; *p* <  0.001) and multiple instillations (RR, 0.61; 95% CI, 0.52–0.72; *p* <  0.001) had protective role on recurrence of bladder cancer after RNU. A further meta-analysis in our study showed that there was no significant difference about the efficacy of preventing bladder recurrence between multiple instillations group and single-instillation group (RR, 0.93; 95% CI, 0.26–3.29; *p*=0.912). A comprehensive literature search showed that single-dose intravesical chemotherapy may lower the risk of bladder cancer recurrence compared to no instillation; however, the effect of single-dose instillation on minor or serious adverse events was uncertain [49]. In the study of early single-dose mitomycin C intravesical instillation after robot-assisted radical nephroureterectomy, patients did not show adverse reactions potentially associated with mitomycin C instillation within 30 days [50]. Conversely, multiple mitomycin C instillation for high-risk nonmuscle-invasive bladder cancer may lead to adverse events, such as urinary frequency, incontinence, and urinary tract pain [51]. For intravesical multiple BCG instillation, fever, appetite loss, and malaise were the most frequent in the general condition of adverse events [52]. Therefore, single instillation may not only improved quality of life for patients with lower potential adverse events, but also reduced living stress of patients due to reduced financial sharing, compared with multiple instillations. Based on consideration of inherent heterogeneity among intravesical drugs or regimen, large-scale multicenter studies would facilitate the unraveling of the mystery with caution. (3). In addition to postoperative intravesical instillation, would clinicians apply intraoperative intravesical chemotherapy for UUT-UC patients during surgery? After searching electronic databases, a meta-analysis identifying two related studies was conducted, and the results suggested that the intraoperative instillation group was associated with a decrease in the risk of bladder recurrence compared with the postoperative instillation group (RR, 2.64; 95% CI, 1.20–5.83; *p*=0.016) [53, 54]. Moriarty et al. proposed the safety of intraoperative instillation, which can ensure the earliest and safest delivery method while eliminating the concerns of delaying instillation due to extravasation [55]. Despite the limited number of studies, intraoperative instillation may be an alternative option for patients who cannot tolerate the adverse reactions of postoperative instillation. (4). Which instillation drugs selected by clinicians are most effective for reducing intravesical recurrence? The ODMIT-C trial demonstrated that intravesical instillation of MMC could significantly reduce the incidence of bladder recurrence [18]. A prospective randomized phase II study in Japan suggested that the intravesical instillation of THP appears to be effective [23]. Another study found that no statistically significant differences were observed in the bladder recurrence rates among the MMC group, EPB group, and control group [15]. Since network meta-analyses are used to analyze multiple therapies synchronously by integrating direct and indirect evidence, we combined 9 eligible studies that included 5 different drugs and performed a network meta-analysis. The results demonstrated that the most effective drug was THP (89.2%), followed by BCG (83.5%), according to the SUCRA value rankings of IVRFS. Therefore, BCG combined with THP may be a promising instillation regimen, which warrants further investigation for verification.

Since there is little research on Northeast China, our retrospective cohort study was conducted to investigate intravesical chemotherapy in preventing intravesical recurrence after RNU. However, some limitations affecting the results of this study are listed as follows. As a single-center study, the retrospective nature of different surgical techniques and experiences by multiple surgeons had inherent potential for selection bias. We enrolled patients who were diagnosed between 2006 and 2017. However, the clinical experience, treatment regimen, and surgical skill may change over time, which could impact the outcomes. Furthermore, a relatively small cohort of 158 patients may limit the analysis of more risk factors for intravesical recurrence, such as history of hypertension, history of diabetes, history of cerebrovascular disease, history of smoking, history of drinking, marital status, and others. Thus, conclusions about efficacy should be made with caution. On the other hand, direct meta-analysis and network meta-analysis provide comprehensive insights into instillation regimens, including drug selection, opportunity of instillation, course of instillation, and the first instillation time, and yield the most up-to-date evidence about the value of intravesical instillation in preventing bladder cancer recurrence after RNU. However, most studies were retrospective with their own limitations, such as selection bias. Broad heterogeneity exists in the administration schedules of intravesical instillation, including drug type, dose, and drug retention time, which impedes us from drawing unequivocal conclusions. The small number (less than 5) of several meta-analyses limits the robustness of the results. The evidence extracted from eligible trials was limited in determining whether intravesical instillation effects vary according to individual characteristics, including age, sex, ethnicity, and tumor grade. Therefore, further large-scale, multicenter, prospective trials are needed to clarify these findings. Furthermore, causative genetic mutations of UUT-UC should be identified by whole-exome sequencing or whole-genome sequencing to identify pathogenicity factors. Drugs specific for pathogenic factors could be developed to move toward precision medicine. Multidisciplinary collaboration between pharmacology and molecular biology is needed to drive this process.

## 5. Conclusions

A maintenance schedule of intravesical instillation effectively prevents the recurrence of bladder cancer after RNU and improves the overall survival of UUT-UC patients. Further large, prospective studies are needed to verify its value. Compared with other chemotherapy regimens, THP may be a promising drug with favorable efficacy to prevent bladder recurrence. As the included studies had a moderate risk of bias, the conclusions of the network meta-analysis should be applied cautiously.

## Figures and Tables

**Table 1:** Patient and tumor characteristics.

	Intravesical instillation, no. (%)			Nonintravesical instillation, no. (%)	Total	*p* Value
	EPB	HCPT	BCG			
Gender						0.040
Male	18 (43.9)	21 (63.6)	16 (44.5)	33 (68.7)	88 (55.7)	
Female	23 (56.1)	12 (36.4)	20 (55.5)	15 (31.3)	70 (44.3)	
Age, years						0.135
Less than 69	21 (51.2)	16 (48.5)	13 (36.1)	26 (54.2)	76 (48.1)	
69 or greater	20 (48.8)	17 (51.5)	23 (63.9)	22 (45.8)	82 (51.9)	
Tumor laterality						0.466
Right	26 (63.4)	16 (48.5)	19 (52.8)	23 (47.9)	84 (53.2)	
Left	15 (36.6)	17 (51.5)	17 (47.2)	25 (52.1)	74 (46.8)	
Tumor location						0.311
Calix or pelvis	13 (31.7)	17 (51.5)	13 (36.1)	19 (39.6)	62 (39.2)	
Ureter	26 (63.4)	13 (39.4)	17 (47.2)	25 (52.1)	81 (51.3)	
More than 1	2 (4.9)	3 (9.1)	6 (16.7)	4 (8.3)	15 (9.5)	
Tumor focality						0.112
Unifocal	33 (80.5)	29 (87.9)	23 (63.9)	36 (75.0)	121 (76.6)	
Multifocal	8 (19.5)	4 (12.1)	13 (36.1)	12 (25.0)	37 (23.4)	
Vessel tumor embolus						0.287
Yes	6 (14.6)	5 (15.2)	5 (13.9)	13 (27.1)	29 (18.4)	
None	30 (73.2)	25 (75.8)	29 (80.6)	27 (56.3)	111 (70.2)	
Unclear	5 (12.2)	3 (9.0)	2 (5.5)	8 (16.6)	18 (11.4)	
Tumor stage						0.373
Tis-T_1_	12 (29.3)	6 (18.2)	7 (19.4)	7 (14.6)	32 (20.3)	
T_2_-T_4_	29 (70.7)	27 (81.8)	29 (80.6)	41 (85.4)	126 (79.7)	
Pathologic grade						0.152
Low	11 (26.8)	13 (39.4)	9 (25.0)	8 (16.7)	41 (25.9)	
High	30 (73.2)	20 (60.6)	27 (75.0)	40 (83.3)	117 (74.1)	
Lymph node status						0.474
No	37 (90.2)	32 (97.0)	35 (97.2)	44 (91.7)	148 (93.7)	
N_1_	4 (9.8)	1 (3.0)	1 (2.8)	4 (8.3)	10 (6.3)	
Perineural invasion						0.145
No	40 (97.6)	33 (100.0)	32 (88.9)	44 (91.7)	149 (94.3)	
Yes	1 (2.4)	0 (0.0)	4 (11.1)	4 (8.3)	9 (5.7)	
Surgical approach						0.171
Open	15 (36.6)	15 (45.5)	9 (25.0)	12 (25.0)	51 (32.3)	
Laparoscopic	26 (63.4)	18 (54.5)	27 (75.0)	36 (75.0)	107 (67.7)	

**Table 2:** Univariate and multivariate analysis of factors associated with intravesical recurrence-free survival.

Characteristic	Univariate		Multivariate	
	HR	*p* Value	HR	*p* Value
Gender (female vs. male)	0.643 (0.384–1.077)	0.093		
Age (≥69 vs. <69)	0.768 (0.468–1.260)	0.296		
Tumor laterality (left vs. right)	0.968 (0.589–1.588)	0.896		
Tumor location (ureter/more than 1 vs. Calix or pelvis)	1.040 (0.679–1.592)	0.857		
Tumor focality (multifocal vs. unifocal)	1.047 (0.567–1.934)	0.884		
Vessel tumor embolus (yes vs. no)	1.257 (0.899–1.757)	0.181		
Tumor stage (T_2_-T_4_ vs. Tis-T_1_)	3.005 (1.361–6.633)	0.006	2.691 (1.201–6.032)	0.016
Pathological grade (high vs. low)	2.028 (1.103–3.730)	0.023	1.449 (0.773–2.716)	0.247
Lymph node status (N_1_ vs. N_0_)	1.350 (0.540–3.380)	0.521		
Perineural invasion (yes vs. No)	3.141 (1.107–8.915)	0.032	2.961 (1.036–8.458)	0.043
Surgical approach (laparoscopic vs. open)	0.796 (0.478–1.327)	0.382		
Intravesical instillation (yes vs. no)	0.703 (0.544–0.907)	0.007	0.731 (0.569–0.941)	0.015

## Data Availability

Data are available upon request to the corresponding author.

## Supplementary Materials

Supplementary Materials Supplementary Table 1: PRISMA checklist. Supplementary Table 2: main characteristics of twenty studies included in the meta-analysis. Supplementary Table 3: patient characteristics from eligible studies.
