# Supplementary material for: Assessment of Therapeutic Benefit and Option Strategy on Intravesical Instillation for Preventing Bladder Cancer Recurrence after Radical Nephroureterectomy in Patients with Upper Urinary Tract Urothelial Carcinoma
Source: J Oncol. 2022 May 30;2022:1755368. doi: 10.1155/2022/1755368 (PMC9170511; doi:10.1155/2022/1755368)
Supplement: Supplementary Materials — Supplementary Table 1: PRISMA checklist. Supplementary Table 2: main characteristics of twenty studies included in the meta-analysis. Supplementary Table 3: patient characteristics from eligible studies. [file 1755368.f1.zip › 1755368.f1/Updated Supplementary Table 2.doc]

Supplementary Table 2. Main characteristics of twenty studies included in the meta-analysis^a^.

| First author  (Year) | Region | Study type | No. of Center | Recruitment  period | Total number  (I/C) | Instillation | | | | Recurrence  rate |
| --- | --- | --- | --- | --- | --- | --- | --- | --- | --- | --- |
|  |  |  |  |  |  | Drug and dose | Initiated time | Retained time | Duration |  |
| Katims AB [1]  (2021) | NR | Case-control | Multicenter | 2015-2019 | 429  (316/113) | NR | NR | NR | NR | NR |
| Lai S [2]  (2021) | China | Retrospective  cohort | Multicenter | 2004-2018 | 489  (207/282) | NR | NR | NR | NR | NR |
| Liu J [3]  (2021) | China | Retrospective  cohort | Single | 2004-2019 | 255  (77/178) | THP/MMC | NR | NR | 6-8 times weekly | NR |
| Freifeld Y [4]  (2020) | United States | Retrospective  cohort | Multicenter | 2012-2018 | 137  (111/26)^b^ | MMC 20-40mg/50ml  Gemcitabine 1-2g/50ml | Intraoperative | 60 min | NR | NR |
|  |  |  |  |  |  |  | Postoperative | NR |  |  |
| Li YR [5]  (2020) | China | Case-control | Single | 2012-2016 | 217  (11/206) | EPB/MMC | NR | NR | NR | NR |
| Kim SH [6]  (2020) | Korea | Retrospective  cohort | Single | 2004-2016 | 162  (59/103) | NR | NR | NR | NR | NR |
| Harraz AM [7]  (2019) | Egypt | RCT | Single | 2015-2017 | 74  (36/38) | EPB 50mg/50ml | Within 48 hours | 30 minutes | weekly intervals for 6 weeks to complete a  1-year maintenance | At 6 months  At 1 year  At 2 year |
|  |  |  |  |  |  |  |  |  | Once |  |
| Huang Y [8]  (2019) | China | Retrospective  cohort | Multicenter | 2000-2016 | 270  (229/41) | EPB 30-50mg  125 cases | Within 2 weeks | At least 30 minutes | At least 6 doses weekly | NR |
|  |  |  |  |  |  | THP 30-50mg  89 cases |  |  |  |  |
|  |  |  |  |  |  | MMC 20-40mg  15 cases |  |  | Once |  |
| Noennig B [9]  (2018) | United States | Retrospective  cohort | Single | 2008-2016 | 51  (30/21) | MMC/100ml | Intraoperative | NR | NR | NR |
|  |  |  |  |  |  |  | 24-72h after surgery |  |  |  |
| Yamashita S [10]  (2017) | Japan | Retrospective  cohort | Multicenter | 2012-2013 | 74  (11/63) | THP | NR | NR | NR | NR |
| Jiang G [11]  (2017) | China | Case-control | Single | 2005-2015 | 113  (50/63) | THP | NR | NR | 8 times weekly and followed by monthly for 6-10 months | NR |
| Long X [12]  (2016) | China | Retrospective  cohort | Single | 2004-2012 | 320  (161/159) | EPB 30mg | Within 1-2 weeks | At least 30 minutes | Once | At 1 year  At 3 year |
| Nagashima M [13]  (2013) | Japan | Retrospective  cohort | Single | 2003-2008 | 42  (23/19) | THP 30mg/40ml | After 2 weeks | NR | 5 doses weekly | At 1 year  At 2 year |
|  |  |  |  |  |  |  |  |  |  |  |
| Ito A [14]  (2013) | Japan | RCT | Multicenter | 2005-2008 | 72  (36/36) | THP 30mg/30ml | Within 48 hours | 30 minutes | Once | At 1 year  At 2 year |
| O'Brien T [15]  (2011) | United Kingdom | RCT | Multicenter | 2000-2006 | 220  (105/115) | MMC 40mg/40ml | Within 48 hours | NR | Once | At 1 year |
| Abe T [16]  (2010) | Japan | Case-control | Multicenter | 1990-2004 | 70  (32/38) | EPB/BCG | NR | NR | NR | NR |
| Wu WJ [17]  (2010) | Taiwan | Retrospective  cohort | Single | 1985-2007 | 196  (31/27/138) | EPB 20mg/20ml | Within 2 weeks | At least 1 hour | 6-8 times | At 10 year |
|  |  |  |  |  |  | MMC 10mg/20ml |  |  |  |  |
| Miao M [18]  (2009) | China | Case-control | Single | 1997-2007 | 227  (166/61) | MMC 30mg/50ml  HCPT 30mg/50ml | Within 48 hours or  after 2 weeks | 1 hour | 10 times | NR |
| Sakamoto N [19]  (2001) | Japan | RCT | Multicenter | 1993-1996 | 25  (13/12) | MMC 20mg/10ml  ＋  Ara-C(200mg/10ml) | Within 1-2 weeks | 2 hours | 2 weekly,  5 fortnightly,  21 monthly  28 times | At 1 year  At 2 year |
| Our study | China | Retrospective  cohort | Single | 2006-2018 | 158  (41/33/36/48) | EPB 30mg/50ml | After two weeks | At least half an hour | 8 times | At 1 year  At 3 year  At 5 year |
|  |  |  |  |  |  | HCPT 10mg/50ml |  |  |  |  |
|  |  |  |  |  |  | BCG 120mg/50ml |  |  |  |  |

^a^ BCG, Bacillus Calmette-Guerin; C, control group; EPB, epirubicin; HCPT, hydroxycamptothecin; I, instillation group; MMC, mitomycin C; NR, not reported; RUC, removal of the urethral catheter; THP, pirarubicin.

^b^ I, intraoperative instillation group; C, postoperative instillation group.

**References**

[1]	A. B. Katims, R. Say, I. Derweesh et al., "Risk Factors for Intravesical Recurrence after Minimally Invasive Nephroureterectomy for Upper Tract Urothelial Cancer (ROBUUST Collaboration)," *The Journal of urology,* vol. 206, no. 3, pp. 568-576, 2021.

[2]	S. Lai, X. Long, P. Wu et al., "Developing a nomogram for predicting intravesical recurrence after radical nephroureterectomy: a retrospective cohort study of mainland Chinese patients," *Japanese journal of clinical oncology,* vol. 51, no. 7, pp. 1132-1141, 2021.

[3]	J. Liu, P. Wu, S. Lai et al., "Preoperative Monocyte-to-lymphocyte Ratio Predicts for Intravesical Recurrence in Patients With Urothelial Carcinoma of the Upper Urinary Tract After Radical Nephroureterectomy Without a History of Bladder Cancer," *Clinical genitourinary cancer,* vol. 19, no. 3, pp. e156-e165, 2021.

[4]	Y. Freifeld, R. Ghandour, N. Singla et al., "Intraoperative prophylactic intravesical chemotherapy to reduce bladder recurrence following radical nephroureterectomy," *Urologic oncology*, vol. 38, pp. 737.e11-737.e16, 2020.

[5]	Y. R. Li, K. J. Yu, Y. H. Chang et al., "Predictors of Intravesical Recurrence After Radical Nephroureterectomy and Prognosis in Patients with Upper Tract Urothelial Carcinoma," *Cancer management and research,* vol. 12, no. pp. 7439-7450, 2020.

[6]	S. H. Kim, W. S. Park, B. Park et al., "Identification of Significant Prognostic Tissue Markers Associated with Survival in Upper Urinary Tract Urothelial Carcinoma Patients Treated with Radical Nephroureterectomy: A Retrospective Immunohistochemical Analysis Using Tissue Microarray," *Cancer research and treatment,* vol. 52, no. 1, pp. 128-138, 2020.

[7]	A. M. Harraz, M. El-Shabrawy, A. R. El-Nahas, H. El-Kappany, Y. Osman, "Single Versus Maintenance Intravesical Chemotherapy for the Prevention of Bladder Recurrence after Radical Nephroureterectomy for Upper Tract Urothelial Carcinoma: A Randomized Clinical Trial," *Clinical genitourinary cancer*, vol. 17, pp. e1108-e1115, 2019.

[8]	Y. Huang, J. Cen, Z. Liu et al., "A Comparison of Different Prophylactic Intravesical Chemotherapy Regimens for Bladder Cancer Recurrence After Nephroureterectomy for Primary Upper Tract Urothelial Carcinomas: A Retrospective 2-center Study," *Technology in cancer research & treatment*, vol. 18, pp. 1533033819844483, 2019.

[9]	B. Noennig, S. Bozorgmehri, R. Terry, B. Otto, L. M. Su, P. L. Crispen, "Evaluation of Intraoperative Versus Postoperative Adjuvant Mitomycin C with Nephroureterectomy for Urothelial Carcinoma of the Upper Urinary Tract," *Bladder cancer*, vol. 4, pp. 389-394, 2018.

[10]	S. Yamashita, A. Ito, K. Mitsuzuka et al., "Efficacy of early ureteral ligation on prevention of intravesical recurrence after radical nephroureterectomy for upper urinary tract urothelial carcinoma: a prospective single-arm multicenter clinical trial," *Japanese journal of clinical oncology,* vol. 47, no. 9, pp. 870-875, 2017.

[11]	Jiang G, Wang H, Wang L et al., "Systemic inflammatory responses predict survival outcomes and male gender predicts metachronous bladder cancer of upper tract urothelial carcinoma," *International Journal of Clinical and Experimental Medicine*, vol. 10, no. pp. 8094-8101, 2017.

[12]	X. Long, X. Zu, Y. Li et al., "Epidermal Growth Factor Receptor and Ki-67 as Predictive Biomarkers Identify Patients Who Will Be More Sensitive to Intravesical Instillations for the Prevention of Bladder Cancer Recurrence after Radical Nephroureterectomy," *PloS one*, vol. 11, pp. e0166884, 2016.

[13]	M. Nagashima, A. Araki, M. Yanagisawa et al., "[Effect of prophylactic intravesical instillation of pirarubicin to prevent recurrent bladder tumors following nephroureterectomy for upper urinary tract cancer]," *Hinyokika kiyo. Acta urologica Japonica,* vol. 59, no. 4, pp. 213-216, 2013.

[14]	A. Ito, I. Shintaku, M. Satoh et al., "Intravesical seeding of upper urinary tract urothelial carcinoma cells during nephroureterectomy: an exploratory analysis from the THPMG trial," *Japanese journal of clinical oncology*, vol. 43, pp. 1139-1144, 2013.

[15]	T. O’Brien, E. Ray, R. Singh, B. Coker, R. Beard, "Prevention of bladder tumours after nephroureterectomy for primary upper urinary tract urothelial carcinoma: a prospective, multicentre, randomised clinical trial of a single postoperative intravesical dose of mitomycin C (the ODMIT-C Trial)," *European urology*, vol. 60, pp. 703-710, 2011.

[16]	T. Abe, N. Shinohara, T. Harabayashi et al., "Pathological characteristics and clinical course of bladder tumour developing after nephroureterectomy," *BJU international,* vol. 105, no. 8, pp. 1102-1106, 2010.

[17]	W. J. Wu, H. L. Ke, Y. H. Yang, C. C. Li, Y. H. Chou, C. H. Huang, "Should patients with primary upper urinary tract cancer receive prophylactic intravesical chemotherapy after nephroureterectomy," *The Journal of urology*, vol. 183, pp. 56-61, 2010.

[18]	M. Miao, C. Z. Kong, Z. H. Li, X. K. Liu, and Z. X. Sun, "[The clinical study for reducing bladder cancer recurrence after surgical treatment for renal pelvic carcinoma]," *Zhonghua wai ke za zhi [Chinese journal of surgery],* vol. 47, no. 10, pp. 728-730, 2009.

[19]	N. Sakamoto, S. Naito, J. Kumazawa et al., "Prophylactic intravesical instillation of mitomycin C and cytosine arabinoside for prevention of recurrent bladder tumors following surgery for upper urinary tract tumors: a prospective randomized study," *International journal of urology : official journal of the Japanese Urological Association*, vol. 8, pp. 212-216, 2001.
