# Supplementary material for: Assessment of Therapeutic Benefit and Option Strategy on Intravesical Instillation for Preventing Bladder Cancer Recurrence after Radical Nephroureterectomy in Patients with Upper Urinary Tract Urothelial Carcinoma
Source: J Oncol. 2022 May 30;2022:1755368. doi: 10.1155/2022/1755368 (PMC9170511; doi:10.1155/2022/1755368)
Supplement: Supplementary Materials — Supplementary Table 1: PRISMA checklist. Supplementary Table 2: main characteristics of twenty studies included in the meta-analysis. Supplementary Table 3: patient characteristics from eligible studies. [file 1755368.f1.zip › 1755368.f1/Updated Supplementary Table 3.doc]

Supplementary Table 3. Patient characteristics from eligible studies^a^.

| First author  (Year) | Age  (Years) | Gender  (M/F) | Tumor Stage  (≤T1/≥T2) | Tumor Grade | | Tumor site  (Calix or Pelvis/Ureter/Both) | Follow up  (Months) |
| --- | --- | --- | --- | --- | --- | --- | --- |
|  |  |  |  | High/Low | G3/G2/G1 |  |  |
| Katims AB [1]  (2021) | 69.3±9.9 | NR | NR | NR | NR | NR | 20.5±17.8 |
| Lai S [2]  (2021) | 64.0 (57.0-70.0) | 314/175 | 179/310 | 227/262 | - | 291/198/0 | 52.0 (37.0-74.0) |
| Liu J [3]  (2021) | 69.0 (61.0-75.0) | 124/131 | 139/116^b^ | 188/67 | - | 133/122/0 | 43.9 (19.3-82.8) |
| Freifeld Y [4]  (2020) | 68.4±11.1 | 98/39 | 90/47 | 94/27 | - | 61/52/18 | 8.0 (1.0-55.0) |
| Li YR [5]  (2020) | 70.0 (34.0-90.0) | 79/138 | 119/98 | 164/53 | - | 129/66/22 | 42.0 (1.2-83.3) |
| Kim SH [6]  (2020) | 66.5±10.4 | 119/43 | 33/124 | - | 92/55/5 | 65/63/33 | 53.4 (3.6-176.5) |
| Harraz AM [7]  (2019) | SIC 63.6±10.2  MIC 59.9±10.2 | 67/7 | 48/26 | NR | NR | 17/25/32 | SIC 18.5 (12.0-38.0)  MIC 19.5 (12.0-36.0) |
| Huang Y [8]  (2019) | 64.0 (56.0-72.0) | 179/91 | 111/159 | 181/89 | - | 167/88/15 | 27.5 (10.8-52.8) |
| Noennig B [9]  (2018) | I-I 71.5±10.9  P-I 72.5±9.0 | 39/12 | 32/19 | 35/16 | - | NR | I-I 22.0 (NR)  P-I 12.0 (NR) |
| Yamashita S [10]  (2017) | 71.0 (55.0-86.0) | 44/30 | 30/44 | - | 34/37^c^ | 45/27/2 | 24.0 (9.0-45.0) |
| Jiang G [11]  (2017) | 64.9±10.2 | 76/37 | 64/49 | 76/37 | - | 58/45/10 | 29.0 (2.0-113.0) |
| Long X [12]  (2016) | 61.4±8.3 | 233/87 | 109/211 | - | 91/299^c^ | 213/107 | 52.0 (38.0-68.0) |
| Nagashima M [13]  (2013) | I 69.0 (58.0-81.0)  C 71.0 (42.0-81.0) | 28/14 | 12/30 | - | 10/20/12 | 22/15/5 | 9.0 (3.0-143.0) |
| Ito A [14]  (2013) | NR | 43/29 | 39/33 | 33/39 | - | 40/29/3 | 24.9 (2.6-39.3) |
| O’Brien T [15]  (2011) | I 70.0 (44.0-87.0)  C 71.0 (36.0-90.0) | NR | 139/93 | 18/214 | - | NR | 12.0 (NR) |
| Abe T [16]  (2010) | 66.0 (39.0-87.0) | 55/19 | 73/1 | 15/59 | - | 40/29/5 | 50.5 (12.0-155.0) |
| Wu WJ [17]  (2010) | 65.0 (23.0-86.0) | 92/104 | 86/110 | 115/81 | - | 64/95/0 | I1 57.5 (12.0-161.0)  I2 61.7 (13.0-138.0)  C 54.0 (12.0-182.0) |
| Miao M [18]  (2009) | 62.0 (NR) | 126/101 | 96/131 | 142/85 | - | NR | 58.0 (NA) |
| Sakamoto N [19]  (2001) | I 69.4 (55.0-85.0)  C 69.3 (59.0-80.0) | 16/9 | 25/0 | - | 3/18/4 | NR | 45.0 (6.0-65.0) |
| Our study | 69.0 (59.0-80.0) | 88/70 | 32/126 | 117/41 | - | 62/81/15 | 29.2 (2.3-122.1) |

^a^ C, control group; F, female; I, instillation group; I-I, intraoperative instillation group; IQR, interquartile range; L, left; M, male; MIC, multiple intravesical instillation; NR, not reported; P-I, postoperative instillation group; SIC, single intravesical instillation.[1]

^b^ <T3/≥T3

^c^ G3/G1+G2

**References**

[1]	A. B. Katims, R. Say, I. Derweesh et al., "Risk Factors for Intravesical Recurrence after Minimally Invasive Nephroureterectomy for Upper Tract Urothelial Cancer (ROBUUST Collaboration)," *The Journal of urology,* vol. 206, no. 3, pp. 568-576, 2021.

[2]	S. Lai, X. Long, P. Wu et al., "Developing a nomogram for predicting intravesical recurrence after radical nephroureterectomy: a retrospective cohort study of mainland Chinese patients," *Japanese journal of clinical oncology,* vol. 51, no. 7, pp. 1132-1141, 2021.

[3]	J. Liu, P. Wu, S. Lai et al., "Preoperative Monocyte-to-lymphocyte Ratio Predicts for Intravesical Recurrence in Patients With Urothelial Carcinoma of the Upper Urinary Tract After Radical Nephroureterectomy Without a History of Bladder Cancer," *Clinical genitourinary cancer,* vol. 19, no. 3, pp. e156-e165, 2021.

[4]	Y. Freifeld, R. Ghandour, N. Singla et al., "Intraoperative prophylactic intravesical chemotherapy to reduce bladder recurrence following radical nephroureterectomy," *Urologic oncology*, vol. 38, pp. 737.e11-737.e16, 2020.

[5]	Y. R. Li, K. J. Yu, Y. H. Chang et al., "Predictors of Intravesical Recurrence After Radical Nephroureterectomy and Prognosis in Patients with Upper Tract Urothelial Carcinoma," *Cancer management and research,* vol. 12, no. pp. 7439-7450, 2020.

[6]	S. H. Kim, W. S. Park, B. Park et al., "Identification of Significant Prognostic Tissue Markers Associated with Survival in Upper Urinary Tract Urothelial Carcinoma Patients Treated with Radical Nephroureterectomy: A Retrospective Immunohistochemical Analysis Using Tissue Microarray," *Cancer research and treatment,* vol. 52, no. 1, pp. 128-138, 2020.

[7]	A. M. Harraz, M. El-Shabrawy, A. R. El-Nahas, H. El-Kappany, Y. Osman, "Single Versus Maintenance Intravesical Chemotherapy for the Prevention of Bladder Recurrence after Radical Nephroureterectomy for Upper Tract Urothelial Carcinoma: A Randomized Clinical Trial," *Clinical genitourinary cancer*, vol. 17, pp. e1108-e1115, 2019.

[8]	Y. Huang, J. Cen, Z. Liu et al., "A Comparison of Different Prophylactic Intravesical Chemotherapy Regimens for Bladder Cancer Recurrence After Nephroureterectomy for Primary Upper Tract Urothelial Carcinomas: A Retrospective 2-center Study," *Technology in cancer research & treatment*, vol. 18, pp. 1533033819844483, 2019.

[9]	B. Noennig, S. Bozorgmehri, R. Terry, B. Otto, L. M. Su, P. L. Crispen, "Evaluation of Intraoperative Versus Postoperative Adjuvant Mitomycin C with Nephroureterectomy for Urothelial Carcinoma of the Upper Urinary Tract," *Bladder cancer*, vol. 4, pp. 389-394, 2018.

[10]	S. Yamashita, A. Ito, K. Mitsuzuka et al., "Efficacy of early ureteral ligation on prevention of intravesical recurrence after radical nephroureterectomy for upper urinary tract urothelial carcinoma: a prospective single-arm multicenter clinical trial," *Japanese journal of clinical oncology,* vol. 47, no. 9, pp. 870-875, 2017.

[11]	Jiang G, Wang H, Wang L et al., "Systemic inflammatory responses predict survival outcomes and male gender predicts metachronous bladder cancer of upper tract urothelial carcinoma," *International Journal of Clinical and Experimental Medicine*, vol. 10, no. pp. 8094-8101, 2017.

[12]	X. Long, X. Zu, Y. Li et al., "Epidermal Growth Factor Receptor and Ki-67 as Predictive Biomarkers Identify Patients Who Will Be More Sensitive to Intravesical Instillations for the Prevention of Bladder Cancer Recurrence after Radical Nephroureterectomy," *PloS one*, vol. 11, pp. e0166884, 2016.

[13]	M. Nagashima, A. Araki, M. Yanagisawa et al., "[Effect of prophylactic intravesical instillation of pirarubicin to prevent recurrent bladder tumors following nephroureterectomy for upper urinary tract cancer]," *Hinyokika kiyo. Acta urologica Japonica,* vol. 59, no. 4, pp. 213-216, 2013.

[14]	A. Ito, I. Shintaku, M. Satoh et al., "Intravesical seeding of upper urinary tract urothelial carcinoma cells during nephroureterectomy: an exploratory analysis from the THPMG trial," *Japanese journal of clinical oncology*, vol. 43, pp. 1139-1144, 2013.

[15]	T. O’Brien, E. Ray, R. Singh, B. Coker, R. Beard, "Prevention of bladder tumours after nephroureterectomy for primary upper urinary tract urothelial carcinoma: a prospective, multicentre, randomised clinical trial of a single postoperative intravesical dose of mitomycin C (the ODMIT-C Trial)," *European urology*, vol. 60, pp. 703-710, 2011.

[16]	T. Abe, N. Shinohara, T. Harabayashi et al., "Pathological characteristics and clinical course of bladder tumour developing after nephroureterectomy," *BJU international,* vol. 105, no. 8, pp. 1102-1106, 2010.

[17]	W. J. Wu, H. L. Ke, Y. H. Yang, C. C. Li, Y. H. Chou, C. H. Huang, "Should patients with primary upper urinary tract cancer receive prophylactic intravesical chemotherapy after nephroureterectomy," *The Journal of urology*, vol. 183, pp. 56-61, 2010.

[18]	M. Miao, C. Z. Kong, Z. H. Li, X. K. Liu, and Z. X. Sun, "[The clinical study for reducing bladder cancer recurrence after surgical treatment for renal pelvic carcinoma]," *Zhonghua wai ke za zhi [Chinese journal of surgery],* vol. 47, no. 10, pp. 728-730, 2009.

[19]	N. Sakamoto, S. Naito, J. Kumazawa et al., "Prophylactic intravesical instillation of mitomycin C and cytosine arabinoside for prevention of recurrent bladder tumors following surgery for upper urinary tract tumors: a prospective randomized study," *International journal of urology : official journal of the Japanese Urological Association*, vol. 8, pp. 212-216, 2001.
